# Multivariate prediction of motor diagnosis in Huntington's disease: 12 years of PREDICT‐HD

**DOI:** 10.1002/mds.26364

**Published:** 2015-09-04

**Authors:** Jeffrey D. Long, Jane S. Paulsen, PREDICT‐HD Investigators and Coordinators of the Huntington Study Group

**Affiliations:** ^1^ Department of PsychiatryCarver College of Medicine, The University of IowaIowa CityIAUSA; ^2^ Department of BiostatisticsCollege of Public Health, The University of IowaIowa CityIAUSA; ^3^ Department of NeurologyCarver College of Medicine, The University of IowaIowa CityIAUSA; ^4^ Department of PsychologyThe University of IowaIowa CityIAUSA

**Keywords:** Huntington's disease, prognosis, clinical trials methodology/study design, assessment of cognitive disorders/dementia

## Abstract

**Background:**

It is well known in Huntington's disease that cytosine‐adenine‐guanine expansion and age at study entry are predictive of the timing of motor diagnosis. The goal of this study was to assess whether additional motor, imaging, cognitive, functional, psychiatric, and demographic variables measured at study entry increased the ability to predict the risk of motor diagnosis over 12 years.

**Methods:**

One thousand seventy‐eight Huntington's disease gene–expanded carriers (64% female) from the Neurobiological Predictors of Huntington's Disease study were followed up for up to 12 y (mean = 5, standard deviation = 3.3) covering 2002 to 2014. No one had a motor diagnosis at study entry, but 225 (21%) carriers prospectively received a motor diagnosis. Analysis was performed with random survival forests, which is a machine learning method for right‐censored data.

**Results:**

Adding 34 variables along with cytosine‐adenine‐guanine and age substantially increased predictive accuracy relative to cytosine‐adenine‐guanine and age alone. Adding six of the common motor and cognitive variables (total motor score, diagnostic confidence level, Symbol Digit Modalities Test, three Stroop tests) resulted in lower predictive accuracy than the full set, but still had twice the 5‐y predictive accuracy than when using cytosine‐adenine‐guanine and age alone. Additional analysis suggested interactions and nonlinear effects that were characterized in a post hoc Cox regression model.

**Conclusions:**

Measurement of clinical variables can substantially increase the accuracy of predicting motor diagnosis over and above cytosine‐adenine‐guanine and age (and their interaction). Estimated probabilities can be used to characterize progression level and aid in future studies' sample selection. © 2015 The Authors. *Movement* Disorders published by Wiley Periodicals, Inc. on behalf of International Parkinson and Movement Disorder Society

Huntington's disease (HD) is caused by an expanded cytosine‐adenine‐guanine (CAG) repeat at the huntingtin gene.^1^ Huntington's disease involves cognitive and psychiatric impairments,^2^, ^3^ but the clinical diagnosis of disease in terms of manifestation of motor signs is considered a landmark event.^4^

Motor diagnosis is associated with an accelerated longitudinal trajectory of motor impairments^5^ as well as cognitive and functional decline.^6^, ^7^ For this reason, proximity to motor diagnosis is often the basis for indexes used to characterize progression level in premanifest HD.

Huntington's disease observational studies often focus on the progression level at study entry. Proper inferences require accounting for the different ages and CAG expansions of individuals who enter the study. Thus, most progression indexes are based on the use of CAG and age and possibly their interaction to predict time of diagnosis.^8^, ^9^ However, prediction based on CAG and age at entry is far from perfect, leaving open the possibility that additional variables measured at study entry might improve prediction accuracy and provide a more accurate index of disease progression.

The overarching goal of this study is to use the Neurobiological Predictors of Huntington's Disease (PREDICT‐HD) study database to examine whether the accuracy in predicting the risk of motor diagnosis improves when motor, imaging, cognitive, functional, psychiatric, and demographic variables are considered in addition to CAG repeat length and age at study entry. We plan to examine collections of 34, 12, 2, and 0 predictors measured at study entry. The collection of 34 variables was culled from previous research^10^, ^11^ and has representatives of all the aforementioned domains. The set of 12 consists of variables obtained from a typical motor examination and may be the only variables available in certain research or clinical settings, especially if imaging is not possible. The set of 2 is CAG and age, and the 0 set is a reference for comparison. Additional (unplanned) sets might be examined based on the initial results. Little is known about multivariate prediction of motor diagnosis, and predictors might interact in complex ways, might be highly correlated, or might have nonlinear effects. To allow for such possibilities, the machine learning method of random survival forests (RSF) will be used for the analysis.^12^ Random survival forests do not explicitly characterize predictor effects, as in a single regression equation. Therefore, the final goal is to develop a Cox regression model to illustrate potential predictor effects and provide a reference for future research.

## Methods

### Participants

The study comprised 1,078 participants (64% female) from PREDICT‐HD study, which is a longitudinal observational study of prodromal HD.^11^, ^13^, ^14^ Data collection covered 2002 to 2014, and dropout was less than 5% per year. All participants had prior and independent genetic testing for HD and were found to have CAG expansion of 36 or more (mean = 42.49, standard deviation [SD] = 2.69, min = 36, max = 62). Participants underwent detailed motor, imaging, cognitive, psychiatric, and functional evaluations at study entry and annually thereafter. All structural imaging measures were expressed as a ratio of volume to intracranial volume. Detailed variable description is provided in the Supplemental Data. All study procedures were approved by institutional review boards at each participating site, and written informed consent was obtained from all participants.

The mean age at study entry was 39.78 years (SD = 10.39, min = 18.11, max = 75.85), and the mean number of years of education was 14.46 (SD = 2.60). According to the diagnostic confidence level (DCL) of the Unified Huntington's Disease Rating Scale (UHDRS), none of the participants had a motor diagnosis at study entry (ie, all participants had DCL < 4). Time of motor diagnosis was defined as the years in the study until the first occurrence of DCL = 4.

Participants were followed for up to 12 y (mean = 4.78, SD = 3.30), and 225 (21%) had a prospective rating of motor diagnosis. Over the duration of the study, 88 trained examiners performed the motor examination. The mean number of examinations performed per examiner was 49.43 (SD = 61.99). Furthermore, 61% of the participants had the same rater throughout, 24% had two raters, and 10% had three or more raters. Additional details are provided by Paulsen et al.^10^

### Statistical Analysis

The goal was to predict the risk of motor diagnosis, using variables measured at study entry. The outcome was years to first motor diagnosis in the study, which was censored for 79% of the sample (diagnosis occurred sometime after the last observation).

The primary method of analysis was RSF, which is a variant of random forests^15^ for right‐censored data (see Supplemental Data). The RSF begins by drawing a sample of size *N* with replacement from the sample data. This bootstrap sample constitutes the initial node of a recursive regression tree. At the initial node and all subsequent nodes, a random sample of the predictors is selected. For each predictor sampled, all possible binary splits (eg, TMS = 0 vs. TMS > 0) are formed from the data of a node, and the log‐rank statistic is computed that indexes the extent of survival curve differences between the binary splits. The predictor and its split‐value that produce the largest log‐rank statistic are used to partition a parent node into left and right daughter nodes. For the daughter nodes, the splitting process is repeated until a node has a minimum number of unique diagnosed participants, which was set to 12 for all analysis. Each participant in a terminal node has the survival information of time to diagnosis or censoring, and a diagnosis indicator (0 if censored, 1 if diagnosed). This information is used to compute a survival curve and cumulative hazard function (CHF) for the terminal node based on the Nelson‐Aalen estimator. Many trees are grown, and the CHF is averaged over all trees with similar terminal nodes. It is the average CHF that is the estimated survival information for groups of participants with the same predictor profile. The bootstrap sampling and random sampling of predictors at each node tend to de‐correlate the trees, so that the averaging produces relatively accurate predictions.^16^ The RSF requires minimal data assumptions and automatically accounts for nonlinear effects, complex interactions, and high correlations among predictors.^16^

In the analysis, 2,000 trees were grown for each group of predictors. A small amount of data were missing on some of the predictors, and this was handled with dynamic imputation within RSF, using an iterative algorithm.^12^ The 34‐predictor analysis had 10% missing data, which dropped to 3% for the 12‐predictor analysis, and 0% for the 2‐predictor analysis (CAG and age) and the 0‐predictor analysis (there were no missing data for the outcome of time to diagnosis or censoring).

After averaging over the trees, two methods were considered for evaluating the merit (strength) of a predictor: variable importance and minimal depth. Variable importance compares the prediction error of normally grown trees with that of trees in which the daughter nodes are randomly assigned. If a predictor has little merit in predicting survival, the random assignment will have little effect relative to the normal assignment, and variable importance will be small. Conversely, if a predictor has much merit, a large discrepancy will be seen between normal and random node assignment, and the variable importance will be large.^12^

Minimal depth indexes how close to the root node a predictor tends to be among the trees.^17^ The predictor used to split the initial node is the most important in prediction for a tree, and merit decreases for variables as they appear in deeper nodes. Minimal depth indexes the first‐appearance depth of a variable across all trees. Smaller values of minimal depth indicate greater merit.

Four models were planned before the analysis, and two models were unplanned, being specified based on the results of the planned models. Details are provided in Table 1 (The Cox model was considered unplanned, because the number of predictors and nature of effects were unknown before the analysis). Although RSF produces relatively accurate predictions, when it is used for an unplanned analysis, it is vulnerable to the same biases as any variable selection method.^16^ In addition to the variable selection bias, additional bias was introduced by the data imputation, and a trial‐and‐error approach to selecting a minimum of 12 diagnosed participants for the terminal nodes and the number of predictors to randomly sample at each node (which was set to the square root of the number of predictors, rounded up). To help account for bias, cross‐validation was used to assess the performance of the models in predicting observed diagnosis status. In the cross‐validation, a training sample was drawn with replacement from the sample (not to be confused with the RSF bootstrapping), all the models were developed, and predicted probabilities of diagnosis were computed. The predicted values of the training sample were then compared with the observed diagnosis status of the test sample (participants not in the training sample). The process was repeated 200 times, and results were averaged over the replications. The Brier score (*BS*) was computed to compare the training‐predicted probabilities and the test‐observed diagnosis status. *BS* is analogous to the mean squared error in traditional regression. For a particular survival time, the estimate is $BS=\frac{1}{M}\Sigma {\left(Y_{test}-{\hat{\pi }}_{train}\right)}^{2}$, where $Y_{test}$ is the observed diagnosis status in the test data ( $Y_{test}$ = 0 if not diagnosed and $Y_{test}$ = 1 if diagnosed), ${\hat{\pi }}_{train}$ is the predicted probability of diagnosis developed from the training data, and M is the number of participants in the test set. The *BS* ranges from 0% to 100%, and it will be small when the observed diagnosed participants have a high estimated probability of diagnosis accompanied by the undiagnosed participants having a low estimated probability. Thus, smaller values indicate more accurate prediction, with perfection being *BS* = 0% (and worse possible prediction being *BS* = 100%). A pseudo‐*R* ^2^ can be computed that indexes the relative *BS* for two nested models. If *BS_R_* is the value for the reduced model with fewer predictors and *BS_F_* is the value for the full model, then pseudo‐*R* ^2^ = (*BS_R_* − *BS_F_*) / *BS_R_*. Pseudo‐*R* ^2^ does not have the same variance‐accounted‐for interpretation as *R* ^2^ in traditional regression and reflects only the relative size of the prediction error of two nested models.

**Table 1:** Cross‐validation Brier scores and pseudo‐*R* ^2^ values for 5‐year prediction (left) and 10‐year prediction (right)

		5‐Year		10‐Year	
Model	Planned?	Brier (%)	Pseudo‐*R* ^2^ (%)	Brier (%)	Pseudo‐*R* ^2^ (%)
Reference‐0	Yes	16		25	
RSF‐2	Yes	14	14	21	18
RSF‐12	Yes	12	27	17	33
RSF‐34	Yes	11	34	16	36
RSF‐8	No	12	28	17	33
Cox‐8	No	10	35	15	42

The second column (Planned?) denotes whether the model was planned before the analysis. Reference‐0, Kaplan‐Meier estimates (no predictors); RSF, random survival forest; Cox, the Cox regression model; the number of predictors appears after the model type. Pseudo‐*R* ^2^ is relative to the Reference model and is not computed for that model.

Brier score can be biased with right‐censored data. To address potential bias, the statistic was computed using weights reflecting the inverse of the probability of censoring.^18^ Weights were obtained from a Cox regression model with CAG and age as predictors, and the results changed very little among different censoring model choices. All analysis was carried out using the R program for statistical computing.^19^ The randomForestSRC package^20^ was used for the RSF analysis, and the pec package^21^ was used for the cross‐validation.

## Results

**Figure 1:**
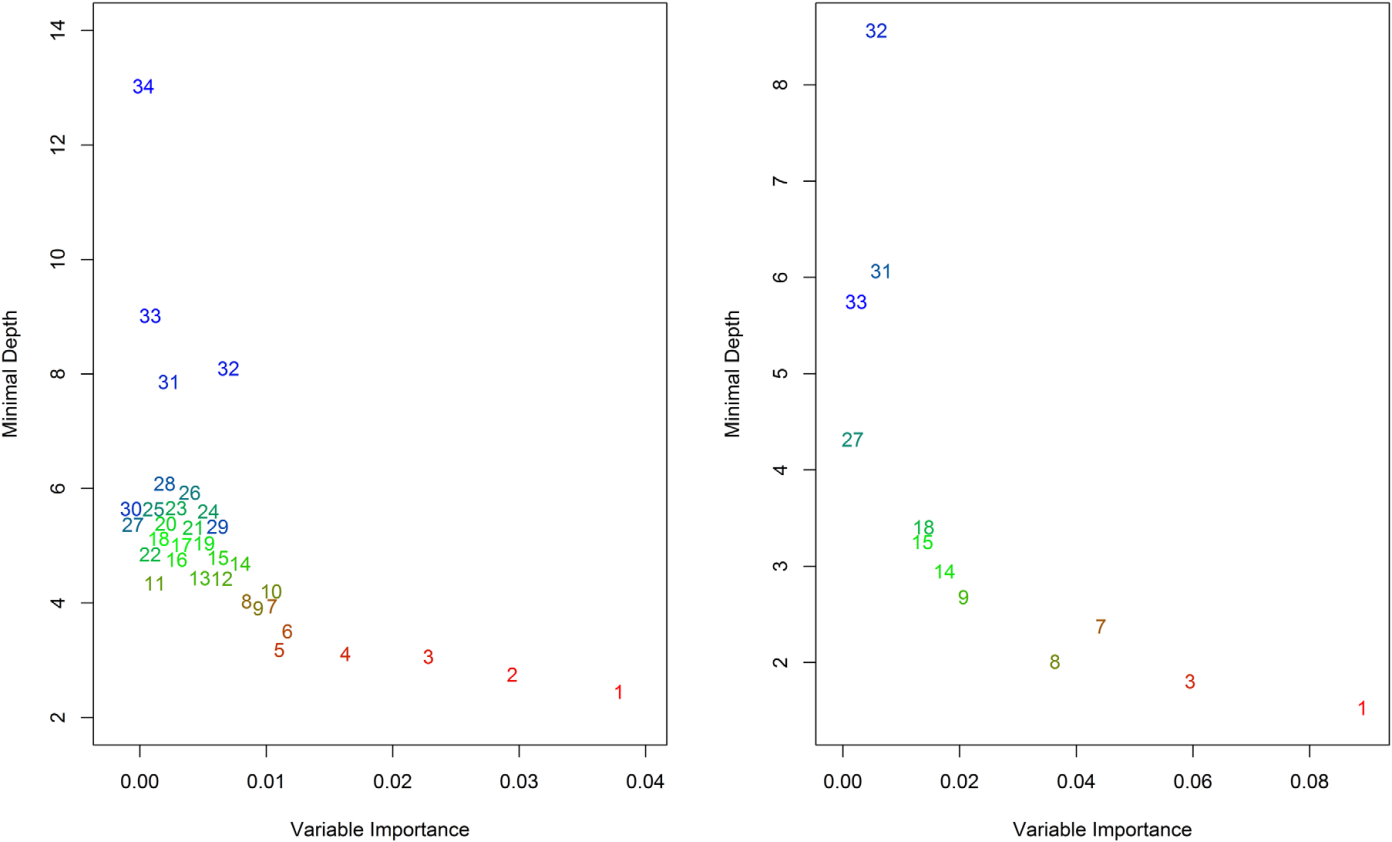
Scatterplot of variable merit (strength of prediction) based on the random survival forest analysis. Minimal depth is shown as a function of variable importance for the group of 34 predictors (left) and 12 predictors (right). Variables are numbered by minimal depth (1 = best), variables in the lower right (red) have the most predictive strength, and variables in the upper left (blue) have the least predictive strength (detailed description of the variables is provided in the Supplemental Data). Key: [1], total motor score from the Unified Huntington's Disease Rating Scale (UHDRS); [2], putamen volume; [3], diagnostic confidence level from the UHDRS; [4], speeded tapping; [5], paced tapping; [6], caudate volume; [7], cytosine‐adenine‐guanine expansion; [8], Symbol Digit Modalities Test; [9], Stroop interference test; [10], accumbens volume; [11], site; [12], University of Pennsylvania Smell Identification Test; [13], Trail Making Test Part A; [14], Stroop color test; [15], Stroop word test; [16], Trail Making Test Part B; [17], emotion recognition test; [18], age (years); [19], Frontal Systems Behavioral Scale—executive subscale; [20], thalamus volume; [21], Symptom Checklist 90—obsessive‐compulsive scale; [22], hippocampus volume; [23], Symptom Checklist 90—Global Severity Index; [24], Frontal Systems Behavioral Rating Scale—disinhibition subscale; [25], Beck Depression Inventory—II; [26], Symptom Checklist 90—anxiety subscale; [27], education (years); [28], Symptom Checklist 90—hostility subscale; [29], Frontal Systems Behavioral Scale—apathy subscale; [30], Symptom Checklist 90—depression subscale; [31], functional activity scale from the UHDRS; [32], total functional capacity from the UHDRS; [33], sex; [34], scanner field strength.

As shown in Table 1, there were four planned models and two unplanned models. Regarding the planned analysis, the no‐predictor model (Reference‐0) was the reference for comparing all of the other models and consisted of the Kaplan‐Meier estimates. The CAG and age model (RSF‐2) represented traditional prediction based on the so‐called burden score in HD.^8^, ^9^ The 34‐predictor model (RSF‐34) had all of the variables, and the 12‐predictor model (RSF‐12) included only the UHDRS variables. The upper portion of Table 1 shows the cross‐validation 5‐y and 10‐y prediction accuracy for the models. As the table shows, all planned models (and unplanned) had greater predictive accuracy (lower error) than Reference‐0, but accuracy was highest for RSF‐34 (eg, 5‐y pseudo‐*R* ^2^ = 34%), followed relatively closely by RSF‐12 (5‐year pseudo‐*R* ^2^ = 27%), and more distantly by RSF‐2 (5‐year pseudo‐*R* ^2^ = 14%). The RSF‐34 was at least twice that of RSF‐2. Figure 1 shows the variable merit scatterplot of minimal depth by variable importance for RSF‐34 (left) and the RSF‐12 (right). Variables with greater merit appear in the lower right (red, smaller numbers) and variables with minimal merit appear in the upper left (blue, larger numbers). The RSF‐34 graph indicates the baseline UHDRS total motor score (TMS) (1) was the best predictor, followed by putamen volume (2), DCL (3), speeded tapping (4), paced tapping (5), caudate volume (6), and CAG expansion (7). The weakest baseline predictors included scanner field strength (34) (1.5 T in most cases), sex (33), the UHDRS total functional capacity (TFC) (32), and Functional Assessment Scale (FAS) (31).

The RSF‐12 graph included four UHDRS cognitive variables, the Symbol Digit Modalities Test (SDMT) (8), and the Stroop word (15), color (14), and interference (9) tests. The TMS (1) was again the best predictor, followed by DCL (3), SDMT (8), CAG (7), the Stroop tests (9, 14, 15), and age (18). The remaining variables (education [27], FAS [31], TFC [32], sex [33]) showed very little merit (weak prediction).

An unplanned eight‐predictor model (RSF‐8) was considered after inspection of Figure 1. The RSF‐12 graph indicates a possible cut‐value for variable reduction below education (27) (other cut‐values are possible). Subsequently, the four weakest variables (education [27], FAS [31], TFC [32], and sex [33]) were excluded for RSF‐8. Finally, an attempt was made to characterize the important effects for the eight‐predictor subset by developing a Cox regression model using additional analysis. Examination of first‐order interactions among all pairs of predictors was performed by randomly assigning daughter nodes to pairs of variables (see Supplemental Data). The largest effect was for CAG × age, followed by CAG × TMS and CAG × DCL, with a relatively large drop thereafter. Because TMS and DCL were highly correlated (Spearman's rho = 0.81), only CAG × age and CAG × TMS were retained for the Cox model.

**Figure 2:**
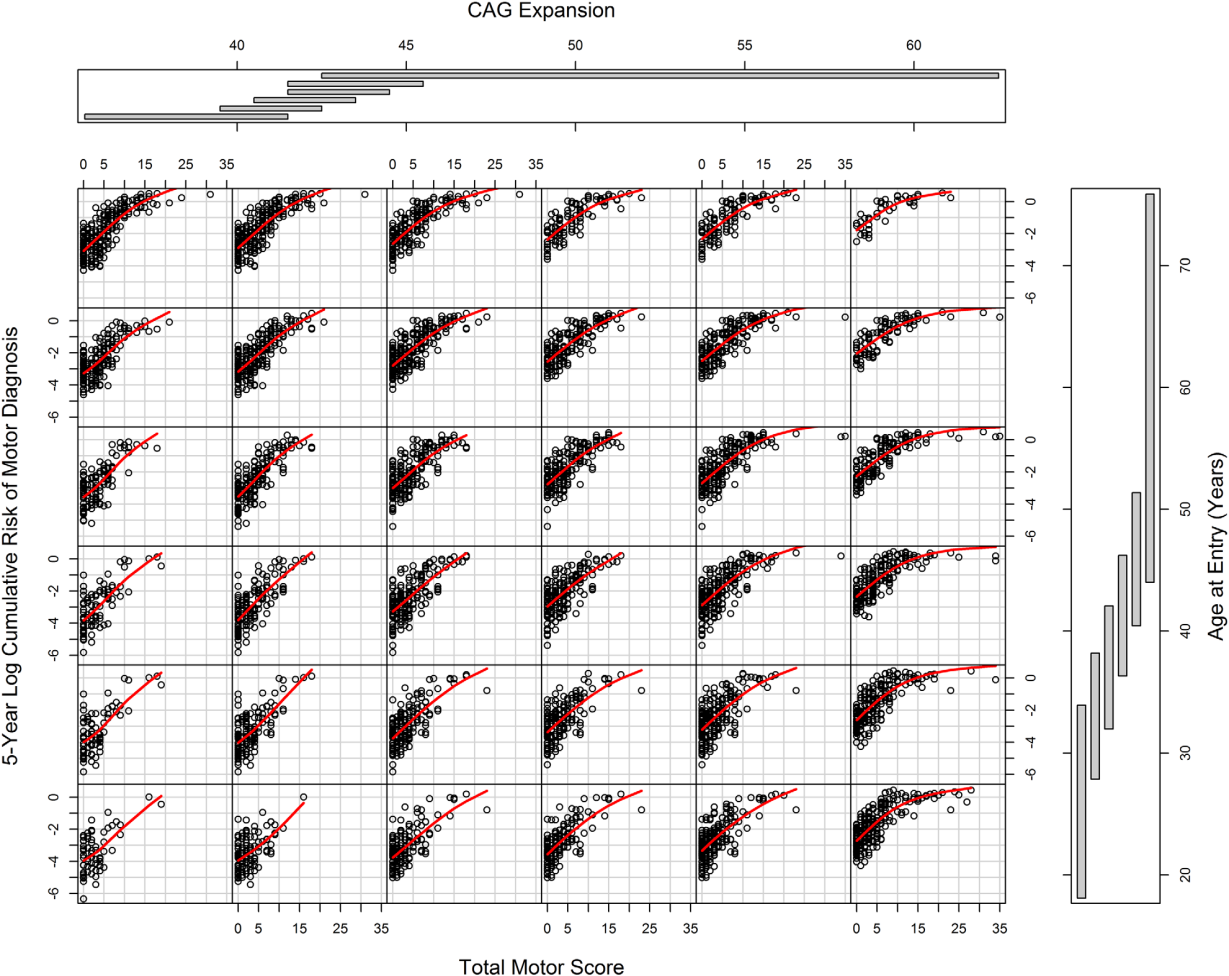
Five‐year log cumulative hazard of motor diagnosis by total motor score, CAG, and age. The slabs of the marginal plots show ranges of the conditioning variables that are used for the interior scatterplot panels. Overlap of the slabs indicates that some participants are used for multiple panels. Smooth curves in the scatterplots (red) are cubic splines. Abbreviation: CAG, cytosine‐adenine‐guanine expansion.

Investigation of nonlinear effects was conducted with graphs such as Figure 2, which shows the relationship between 5‐year log cumulative hazard of diagnosis and TMS conditioning on CAG and age at entry. The layered or slab plots in the margins indicate which CAG and age ranges were used for the interior scatterplots. For example, the extreme lower left scatterplot panel uses participants who had CAG expansion in the range of 36 to 41 and age in the range of 18 to 33. The overlap of the slabs indicates that some participants are used in multiple panels. The slabs for the longest CAGs and the oldest ages are very long, because few participants had this combination, and a wide range had to be used to populate the upper right scatterplot panels. The cubic spline curves of the interior panels illustrate nonlinear effects. For panels that have sufficiently large TMS values (relatively large CAG or older age), the rate of increase decelerates for approximately TMS > 10. Plots for the cognitive variables (not presented) showed much more mild nonlinear trends that were considered negligible and not viable candidate effects for the Cox model. Based on the interaction analysis and the nonlinear TMS effects, the following Cox model was specified:

(1) $$\begin{array}{l} h\left(t\right)=h_{0}\text{exp}\{\beta_{1}DCL_{1}+\beta_{2}DCL_{2}+\beta_{3}DCL_{3}\\ +\beta_{4}TMS+\beta_{5}Color+\beta_{6}Word+\beta_{7}Inter+\beta_{8}SDMT\\ +\beta_{9}CAG+\beta_{10}age+\beta_{11}TMS\times TMS\\ +\beta_{12}CAG\times TMS+\beta_{13}CAG\times age\} \end{array}$$

where h(t) is the time‐dependent hazard, $h_{0}$ is the baseline hazard, DCL_1_ is a dummy code taking the value 1 if DCL = 1 and 0 otherwise, and DCL_2_ and DCL_3_ are similarly defined. The sample‐based estimates for the Cox model along with standard errors (SEs) and *z*‐values are shown in Table 2.

**Table 2:** Cox model sample estimates used to illustrate predictor effects on the risk of motor diagnosis

Effect	Estimate	SE	*z*‐Value
Diagnostic confidence level 1	0.5218	0.2330	2.24
Diagnostic confidence level 2	0.6691	0.2987	2.24
Diagnostic confidence level 3	1.2220	0.3568	3.43
Total motor score (TMS)	0.5723	0.1085	5.27
Stroop color test	−0.0017	0.0081	−0.21
Stroop word test	0.0000	0.0065	0.01
Stroop interference test	−0.0242	0.0102	−2.36
Symbol digit modalities test	−0.0175	0.0088	−1.99
CAG expansion	0.1548	0.0893	1.73
Age at study entry	−0.2802	0.0969	−2.89
TMS × TMS	−0.0039	0.0012	−3.16
CAG × TMS	−0.0097	0.0025	−3.92
CAG × Age	0.0086	0.0023	3.69

The “Estimate” column shows estimates of the *β* parameters in Equation (1) of the text, and “SE” denotes standard error. Diagnostic Confidence Level 1, etc., are dummy codes (1 if in the category and 0 otherwise) representing contrast with the first category (diagnostic confidence level = 0). CAG, cytosine‐adenine‐guanine expansion; TMS, total motor score.

Cross‐validation prediction accuracy for the unplanned models is shown in the bottom portion of Table 1. The RSF‐8 had nearly identical prediction accuracy as RSF‐12, indicating the lack of merit for the four omitted variables. Cox‐8 had the best predictive accuracy of all the models, suggesting that the terms in Equation (1) represented effects important for prediction.

An illustration of the results is provided in Table 3. The RSF‐8 was used to compute estimated probability of motor diagnosis at 2.5, 5, 7.5, and 10 years (at right) for various predictor profiles (at left). The profiles were chosen to represent the spectrum of progression in the PREDICT‐HD sample. Individuals with the same profile have the same predicted probabilities, but predicted probabilities can be computed for every participant.

**Table 3:** Estimated probability of motor diagnosis by year (right) computed using the predictor scores at study entry (left)

	**Predictor profile at baseline**								**Year**			
Profile	CAG	Age	TMS	DCL	SDMT	Inter	Word	Color	2.5	5	7.5	10
A	42	41	1	0	65	46	104	78	0.01	0.05	0.23	0.33
B	42	44	3	1	48	38	118	89	0.02	0.08	0.25	0.42
C	42	53	7	2	38	47	99	68	0.20	0.56	0.72	0.82
D	42	56	20	3	27	18	64	37	0.62	0.83	0.94	0.95

Prediction is based on the random survival forest grown with the 8 variables and using the entire PREDICT‐HD gene–expanded sample (*N* = 1,078).

CAG, cytosine‐adenine‐guanine expansion; Age, age at study entry; TMS, total motor score; DCL, diagnostic confidence level; SDMT, Symbol Digit Modalities Test; Inter, Stroop interference test; Word, Stroop word test; Color, Stroop color test.

## Discussion

The purpose of this study was to investigate whether variables other than CAG repeat length and age (and their interaction) enhanced the prediction of HD motor diagnosis. Such prediction is important because it is the foundation for progression indexes often used to classify participants according to their progression level at study entry.^11^ The PREDICT‐HD database was used to examine the ability of variables collected at study entry to predict the risk of first DCL = 4 up to 12 y. Results based on a machine learning method (RSF) indicate that predictive accuracy was substantially improved by adding variables, consistent with previous findings.^22^, ^23^ To get a sense of the effect size, the 5‐y pseudo‐*R* ^2^ for RSF‐8 (CAG, age, TMS, DCL, Stroop [color, word, and interference], and SDMT) was twice that of RSF‐2 (CAG and age). Therefore, the addition of the six motor and cognitive variables doubled the 5‐y predictive accuracy relative to using just CAG and age (and their interaction). The effect for all 34 variables was even stronger.

The results show that more accurate forecasting information can be obtained by including the UHDRS motor and cognitive variables collected at study entry. Group‐level forecasts can be computed based on baseline predictor profiles (Table 3). The probabilities can be used to index progression level at study entry. For example, in Table 3, Profiles A and B have a low probability of motor diagnosis in the near future (eg, by 5 y) and represent less progression at study entry than Profiles C and D.

One use for the probabilities is for planning clinical trials and observational studies. If a clinical trial is designed for early intervention, then the goal is to recruit individuals who have a relatively long time until motor diagnosis. A low criterion on 5‐y probability can be set, such as ≤0.20, to help ensure that early progressors are selected based on their screening values. For an observational study, the goal might be to recruit a variety of progression levels to maximize the correlation with other variables. Recruitment might target an equal number of individuals on either side of a 5‐y probability of 0.50, for example. Although probabilities can be computed for individuals, the probabilities are at the group level, representing the estimated proportion of individuals who become diagnosed over time. A web‐based HD calculator for computing the estimated probabilities is available from the authors.

The results of the current study are related to the CAG‐Age Product (CAP) burden score previously developed by PREDICT‐HD researchers, which is used to index progression at study entry.^9^ Our approach with RSF is to work directly with diagnosis probability rather than a proxy score, because the probability is more readily interpretable. The cross‐validation results (Table 1) indicate that the RSF probabilities are preferable to those underlying RSF‐2 that represent CAP because of the superior predictive accuracy.

The Cox model (Equation (1) and Table 2) is an attempt to explicitly characterize how the predictors influence the risk of diagnosis. The *z*‐values for Stroop word and color are very close to 0, suggesting that these variables are candidates for omission. The negligible effects are attributable to a high intercorrelation (*r* = 0.76) and a high correlation with interference (*r*s = 0.68, 0.60). No additional cost is incurred in gathering word and color, because they must be administered before interference.^24^

The TMS × TMS effect in Table 2 has a relatively large *z*‐value. To our knowledge, the nonlinear finding is novel. The effect is attributable to a much higher maximum value for TMS than DCL. Once individuals are diagnosed, the DCL no longer tracks progression, whereas the TMS continues to do so. This phenomenon accounts for the log cumulative risk of diagnosis remaining relatively constant as TMS continues to increase (Fig. 2).

We emphasize the group of eight predictors (RSF‐8) in our discussion because it balances effect size and parsimony. Parsimony is especially relevant when the goal is screening individuals for study selection. Huntington's disease is a rare disease (approximately 6/10,000 for whites),^25^ and conducting screenings can be costly because of participant travel.^26^ The eight predictors can be collected at an UHDRS examination without expensive instrumentation or methods. The added striatal volume and tapping measures in the 34‐predictor group appear to be the main reason why RSF‐34 had the best overall predictive accuracy (Fig. 1). A problem with the imaging and tapping variables is the considerable resources required for their collection (magnetic resonance imaging scan and finger‐pressing apparatus). These variables might not be available in all clinical or research contexts and may be too expensive to be used in screening for study selection.

The predictive ability of TMS and DCL shows that earlier motor information predicts later motor status. Because of the inclusion criteria for PREDICT‐HD, DCL at study entry involved categories 0 (normal) to 3 (90%‐98% confident of likely signs of HD). The predictive ability of these categories suggests that DCL can track progression before motor diagnosis. The absence of individuals with DCL = 4 at study entry did not necessarily prevent the inclusion of false negatives or those with apparent motor signs. For example, 25% of participants rated as DCL = 3 at study entry had TMS > 20 (Supplemental Data Fig. S2). The calibration of the TMS relative to the DCL and its applicability to motor diagnosis is a continuing topic of research.

A number of variables showed especially poor prediction, most notably TFC and FAS. Almost all the participants had normal or near‐normal functioning at study entry. Alternative measures of functioning might be more sensitive early in the disease. For example, the World Health Organization Disability Assessment Schedule appears to show a greater variability than TFC before motor diagnosis.^27^, ^28^ Similar to the functional variables, little variability in psychiatric symptoms was seen at study entry, limiting their ability to predict future diagnosis.

Finally, the cross‐validation used in the analysis was an internal validation, and external validation with an independent data set is needed to verify the present results. The initial reduction in predictors from 34 to 12 was preplanned and motivated by practical considerations. Additional reduction to eight predictors was relatively straightforward given the obvious weakness of TFC and FAS. A consequence of the strategy is that the groups of predictors studied here might not be optimal, and the list is by no means complete. Several biofluid and genetic markers,^29^ and behavioral and environmental variables,^30^ for example, show promise for tracking HD progression and would be good candidates for inclusion in future research.

## Author Roles

1. Research Project: A. Conception, B. Organization, C. Execution; 2. Statistical Analysis: A. Design, B. Execution, C. Review and Critique; 3. Manuscript Preparation: A. Writing the First Draft, B. Review and Critique.

J.D.L: 1A, 2A, 2B, 2C, 3A, 3B.

J.S.P: 1A, 1B, 1C, 2A, 2C, 3A, 3B.

## Financial Disclosures

Dr. Long has a consulting agreement with NeuroPhage, LLC. Dr. Paulsen has served on an advisory board for Lundbeck, LLC and has a consulting agreement with ProPhase, LLC.

## Supporting information

Additional Supporting Information may be found in the online version of this article at the publisher's web'zsite.

Supplementary Information

## PREDICT‐HD Investigators, Coordinators, Motor Raters, Cognitive Raters

Isabella De Soriano, Courtney Shadrick, and Amanda Miller (University of Iowa, Iowa City, Iowa, USA);

Edmond Chiu, Joy Preston, Anita Goh, Stephanie Antonopoulos, and Samantha Loi (St. Vincent's Hospital, The University of Melbourne, Kew, Victoria, Australia);

Phyllis Chua, and Angela Komiti (The University of Melbourne, Royal Melbourne Hospital, Melbourne, Victoria, Australia);

Lynn Raymond, Joji Decolongon, Mannie Fan, and Allison Coleman (University of British Columbia, Vancouver, British Columbia, Canada);

Christopher A. Ross, Mark Varvaris, Maryjane Ong, and Nadine Yoritomo (Johns Hopkins University, Baltimore, Maryland, USA);

William M. Mallonee and Greg Suter (Hereditary Neurological Disease Centre, Wichita, Kansas, USA);

Ali Samii, Emily P. Freney, and Alma Macaraeg (University of Washington and VA Puget Sound Health Care System, Seattle, Washington, USA);

Randi Jones, Cathy Wood‐Siverio, and Stewart A. Factor (Emory University School of Medicine, Atlanta, Georgia, USA);

Roger A. Barker, Sarah Mason, and Natalie Valle Guzman (John van Geest Centre for Brain Repair, Cambridge, UK);

Elizabeth McCusker, Jane Griffith, Clement Loy, Jillian McMillan, and David Gunn (Westmead Hospital, Sydney, New South Wales, Australia);

Michael Orth, Sigurd Süβmuth, Katrin Barth, Sonja Trautmann, Daniela Schwenk, and Carolin Eschenbach (University of Ulm, Ulm, Germany);

Kimberly Quaid, Melissa Wesson, and Joanne Wojcieszek (Indiana University School of Medicine, Indianapolis, Indiana, USA);

Mark Guttman, Alanna Sheinberg, Albie Law, and Irita Karmalkar (Centre for Addiction and Mental Health, University of Toronto, Markham, Ontario, Canada);

Susan Perlman and Brian Clemente (UCLA Medical Center, Los Angeles, California, USA);

Michael D. Geschwind, Sharon Sha, Joseph Winer, and Gabriela Satris (University of California, San Francisco, San Francisco, California, USA);

Tom Warner and Maggie Burrows (National Hospital for Neurology and Neurosurgery, London, UK);

Anne Rosser, Kathy Price, and Sarah Hunt (Cardiff University, Cardiff, Wales, UK);

Frederick Marshall, Amy Chesire, Mary Wodarski, and Charlyne Hickey (University of Rochester, Rochester, New York, USA);

Peter Panegyres, Joseph Lee, Maria Tedesco, and Brenton Maxwell (Neurosciences Unit, Graylands, Selby‐Lemnos & Special Care Health Services, Perth, Western Australia, Australia);

Joel Perlmutter, Stacey Barton, and Shineeka Smith (Washington University, St. Louis, Missouri, USA);

Zosia Miedzybrodzka, Daniela Rae, Vivien Vaughan, and Mariella D'Alessandro (Clinical Genetics Centre, Aberdeen, Scotland, UK);

David Craufurd, Judith Bek, and Elizabeth Howard (University of Manchester, Manchester, UK);

Pietro Mazzoni, Karen Marder, and Paula Wasserman (Columbia University Medical Center, New York, New York, USA);

Rajeev Kumar, Diane Erickson, Christina Reeves, and Breanna Nickels (Colorado Neurological Institute, Englewood, Colorado, USA);

Vicki Wheelock, Lisa Kjer, Amanda Martin, and Sarah Farias (University of California, Davis, Sacramento, California, USA);

Wayne Martin, Oksana Suchowersky, Pamela King, Marguerite Wieler, and Satwinder Sran (University of Alberta, Edmonton, Alberta, Canada);

Anwar Ahmed, Stephen Rao, Christine Reece, Alex Bura, and Lyla Mourany (Cleveland Clinic Foundation, Cleveland, Ohio, USA);

### Executive Committee

Principal Investigator Jane S. Paulsen, Jeffrey D. Long, Hans J. Johnson, Thomas Brashers‐Krug, Phil Danzer, Amanda Miller, H. Jeremy Bockholt, and Kelsey Montross.

### Scientific Consultants

Deborah Harrington (University of California, San Diego); Holly Westervelt (Rhode Island Hospital/Alpert Medical School of Brown University); Elizabeth Aylward (Seattle Children's Research Institute); Stephen Rao (Cleveland Clinic); David J. Moser, Janet Williams, Nancy Downing, Vincent A. Magnotta, Hans J. Johnson, Thomas Brashers‐Krug, Jatin Vaidya, Daniel O'Leary, and Eun Young Kim (University of Iowa).

### Core Sections

#### Biostatistics

Jeffrey D. Long, Ji‐In Kim, Spencer Lourens (University of Iowa); Ying Zhang and Wenjing Lu (University of Indiana).

#### Ethics

Cheryl Erwin (Texas Tech University Health Sciences Center); Thomas Brashers‐Krug, Janet Williams (University of Iowa); and Martha Nance (University of Minnesota).

#### Biomedical Informatics

H. Jeremy Bockholt, Jason Evans, and Roland Zschiegner (University of Iowa).
