# Supplementary material for: Multivariate prediction of motor diagnosis in Huntington's disease: 12 years of PREDICT‐HD
Source: Mov Disord. 2015 Sep 4;30(12):1664–72. doi: 10.1002/mds.26364 (PMC4795466; doi:10.1002/mds.26364)
Supplement: Supplementary file 1 — Supplementary Information [file MDS-30-1664-s001.docx]

Supplemental Material

**Multivariate prediction of motor diagnosis in Huntington disease: 12 years of PREDICT-HD**

Supplemental material

Jeffrey D. Long,^1,2^ Jane S. Paulsen, ^1,3,4*^ and the PREDICT-HD Investigators and Coordinators of the Huntington Study Group

^1^Department of Psychiatry, Carver College of Medicine, The University of Iowa, Iowa City, IA, USA

^2^Department of Biostatistics, College of Public Health, The University of Iowa, Iowa City, IA, USA

^3^Department of Neurology, Carver College of Medicine, The University of Iowa, Iowa City, IA, USA

^4^Department of Psychology, The University of Iowa, Iowa City, IA, USA

**Variable descriptions**

**CAG** = cytosine-adenine-guanine expansion.

**Age** = age at study entry.
**Female** = gender.

**Education** = year of education at study entry.

**Putamen** = putamen volume divided by baseline intra-cranial volume (ICV).

**Caudate** = caudate volume divided by baseline ICV.

**Thalamus** = thalamus volume divided by baseline ICV.

**Accumben** = accumbens volume divided by baseline ICV.

**Hippo** = hippocampus volume divided by baseline ICV.

**FS** = scanner field strength (1.5 or 3T).

**Site** = coding for study site.

**TMS** = total motor score from the Unified Huntington’s Disease Rating Scale (UHDRS). Standardized ratings of oculomotor function, dysarthria, chorea, dystonia, gait and postural stability.^1^

**DCL** = diagnostic confidence level from the UHDRS. Standardized ratings of degree to which the examiner thinks the participant has unequivocal motor signs of Huntington disease (HD).^1^

**TapP** = paced tapping or time production. Participants were presented with a 1.8 Hz tone and were instructed to tap along with it when ready. After 11 more presentations of the tone, the tone stopped, and participants attempted to continue to tap at the same pace for 31 more taps. The variable analyzed is the reciprocal of the standard deviation of the intertap interval for an alternating thumbs trial (smaller values indicate worse performance) over five trials.^2^

**TapS** = speeded tapping. Finger tapping speed was assessed by calculating the mean intertap interval of five 10-second trials of tapping as quickly as possible with the nondominant finger (smaller values indicate better performance).^2^

**TFC** = total functional capacity from the UHDRS. A list of independent and common daily tasks that can be accomplished.^1^

**FAS** = functional activity scale from the UHDRS.^1^

**SDMT** = Symbol Digit Modalities Test. The SDMT is an adaptation of the Wechsler Digit Symbol subtest that measures working memory, complex scanning, and processing speed.^3, 4^ Participants use a key presented at the top of the test page to match symbols with numbers presented in horizontal rows. The task requires that the participant fill in the appropriate symbols below the matching numbers as quickly as possible. Raw scores indicate the number of items correctly completed in 90 seconds.^5^

**Color** = Stroop Color and Word Test – color condition. The Stroop Color and Word Test consists of three 45-second trials.^6^ The first two trials (color identification and word reading) measure basic attention. In the first trial, participants must correctly identify the color of ink patches on a stimulus card. In the second trial, participants read the names of colors printed in black ink. In the third trial, the interference trial, participants must consistently inhibit an overlearned response by identifying the color of ink (red, green, blue) that the stimulus color words are printed in rather than reading the word aloud. Raw scores indicate the number of items correctly completed per trial.^7^

**Word** = Stroop Color and Word Test – word condition. The Stroop Color and Word Test consists of three 45-second trials.^6^ The first two trials (color identification and word reading) measure basic attention. In the first trial, participants must correctly identify the color of ink patches on a stimulus card. In the second trial, participants read the names of colors printed in black ink. In the third trial, the interference trial, participants must consistently inhibit an overlearned response by identifying the color of ink (red, green, blue) that the stimulus color words are printed in rather than reading the word aloud. Raw scores indicate the number of items correctly completed per trial.^7^

**Inter** = Stroop Color and Word Test – interference condition. The Stroop Color and Word Test consists of 3 45-second trials.^6^ The first two trials (color identification and word reading) measure basic attention. In the first trial, participants must correctly identify the color of ink patches on a stimulus card. In the second trial, participants read the names of colors printed in black ink. In the third trial, the interference trial, participants must consistently inhibit an overlearned response by identifying the color of ink (red, green, blue) that the stimulus color words are printed in rather than reading the word aloud. Raw scores indicate the number of items correctly completed per trial.^7^

**Smell** = University of Pennsylvania Smell Identification Test (UPSIT). The smell identification test is a multiple-choice measure of olfactory recognition. Participants scratched a scented patch in a test booklet and identified the corresponding scent label from four multiple choice options.^8^ Some participants completed the full four-booklet version of the UPSIT. Others completed an abbreviated, 20-item version. The percentage of correctly identified scents was analyzed.^8^

**TrA** = Trail Making Test, Part A. In TrA, participants draw lines connecting numbered circles as quickly as possible. Raw scores indicate the number of seconds required to complete each test.^9, 10^

**TrB** = Trail Making Test, Part B. In TrB, participants alternate between connecting numbered and lettered circles according to ascending, alphabetical order (i.e., 1-A, 2-B, 3-C, etc.). Raw scores indicate the number of seconds required to complete each test.^9, 10^

**Emotions** = emotion recognition test. Emotion recognition was assessed by two emotion-labeling tasks.^11^ One of the tasks employed static photographs of human faces, while the other used the same stimuli with simulated movement.^12^ In both tasks, participants were asked to identify the emotion displayed by a target face. In the static condition, an expression of moderate intensity was presented for one second. The options were fear, disgust, happy, sad, surprise, anger, and neutral. In the simulated movement condition, an expression of mild intensity presented for 500 milliseconds transformed into an expression of moderate intensity for 500 milliseconds. The variables analyzed for each are the number of negative emotions correctly identified.^11, 13^

**OC** = Symptom Checklist 90 – obsessive compulsive scale – companion rating scale. A 90-item assessment taking 15 minutes to administer, with this subscale focusing on obsessive compulsive disorders as rated by companions.^14^

**ExecFunc** = Frontal Systems Behavioral Scale – executive subscale – companion rating scale. Part of a 46-item behavior rating scale focusing on abstraction, problem solving and hypothesis generation as rated by a companion focusing with dorsolateral prefrontal circuitry.^15^

**Apathy** = Frontal Systems Behavioral Scale – apathy subscale – companion rating scale. Part of a 46-item behavior rating scale associated with anterior cingulate circuitry.^15^

**GSI** = Symptom Checklist 90 – Global Severity Index – companion rating scale. A 90-item assessment taking 15 minutes to administer. Global severity is one of the three major indices.^14^

**Depress** = Symptom Checklist 90 – depression subscale – companion rating scale. A 90-item assessment taking 15 minutes to administer, with this subscale focusing on depressive symptoms as rated by companions.^14^

**Anxiety** = Symptom Checklist 90 – anxiety subscale – companion rating scale. A 90-item assessment taking 15 minutes to administer, with this subscale focusing on anxiety as rated by companions.^14^

**BDI** = Beck Depression Inventory–II. A 21-question inventory to measure the severity of depression.^16^

**Disin** = Frontal Systems Behavioral Rating Scale – disinhibition subscale – companion rating scale. Part of a 46-item scale associated with orbitofrontal circuitry.^15^

**Hostil** = Symptom Checklist 90 – hostility subscale – companion rating scale. A 90-item assessment taking 15 minutes to administer with this subscale focusing on outward hostility toward others as rated by companions.^14^

**Statistical Analysis: Random Survival Forest**

**Figure S1**. Illustration of a single regression tree in random survival forest.


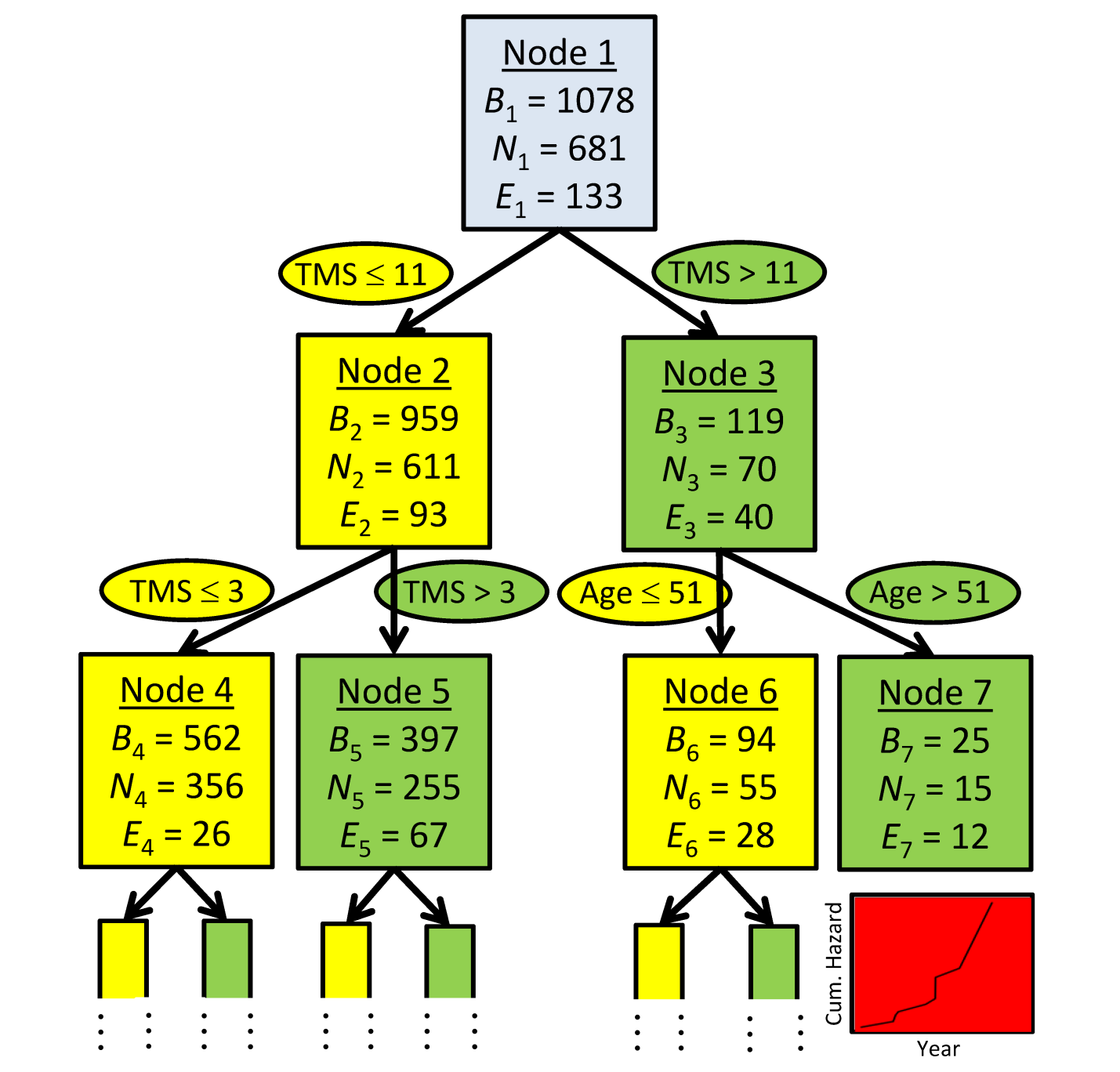
 *B*_1_, initial bootstrap sample size; *N*_1_, number of unique subjects; *E*_1_, number of unique events (diagnosis); TMS, total motor score; Cum. Hazard, cumulative hazard. The vertical ellipses at lower left indicate the daughter nodes continue to be divided to the terminal nodes.

The primary method of analysis was random survival forests (RSF), which is a variant of random forests^17^ for right-censored data. Figure S1 is used to illustrate the RSF method. RSF begins by drawing a sample with replacement from the sample data of size *N*. This bootstrap sample constitutes the initial node (Node 1) of a recursive regression tree. In the Node 1 box (top gray box), *B*_1_ is the size of the bootstrap sample (*B*_1_ = *N*), *N*_1_ is the number of unique subjects in the sample (sampling is with replacement), and *E*_1_ is the number of unique individuals who received a motor diagnosis in the bootstrap sample. At Node 1 and all subsequent nodes, a random sample of the predictors is selected. For each predictor sampled, all possible binary splits (e.g., TMS = 0 vs TMS > 0, etc.) are formed from the data of a node, and the log-rank statistic is computed that indexes the extent of survival curve differences between the binary splits. The variable and split value that produce the largest log-rank statistic are used to partition a parent node into left (yellow) and right (green) daughter nodes. For Node 1, this is TMS with a cut-value of 11 depicted by the ellipses next to the arrows (TMS ≤ 11 and TMS > 11). The split produces a left node (Node 2) with *B*_2_ = 959 of the original bootstrap sample (*N*_2_ = 611 of the original unique individuals with *E*_2_ = 93 events), and a relatively low proportion of diagnosed (93/611 = 0.15). The right node (Node 3) has the remaining *B*_3_ = 119 (*B*_1_ = *B*_2_ + *B*_3_) bootstrap cases and a higher proportion of diagnosed (40/70 = 0.57). Thus, the splitting process tends to discriminate survival information among groups. For the daughter nodes, the splitting process is repeated (no new bootstrap samples are taken) until a node has a minimum number of unique diagnosed. The minimum number was set to 12 for all analysis. Node 7 is a terminal node because *E*_7_ = 12 and daughter nodes would have less than 12 diagnosed (so, Node 7 is not further split). Each terminal node has a subset of the initial bootstrap sample, *B*_7_ = 25 in the case of Node 7 (with *N*_7_ = 15 being unique). Each subject in a terminal node has the survival information of time to diagnosis or censoring, and a diagnosis indicator (0 if censored, 1 if diagnosed). This information is used to compute a survival curve and cumulative hazard function (CHF) for the terminal node based on the Nelson-Aalen estimator. The CHF for Node 7 is depicted by the red graph at the extreme lower right, and represents the cumulative risk of diagnosis for subjects with TMS > 11 and Age > 51 for this one tree. Terminal nodes are eventually determined for all daughters on the left side of Figure S1. Figure S1 depicts a single tree, but many trees are grown in RSF (e.g., 2000). The CHF is averaged over all trees with similar terminal nodes, and it is the average CHF (and corresponding survival curve) that is the survival information for subjects with the same predictor profile. The bootstrap sampling and random sampling of predictors at each node tends to de-correlate the trees, so that the averaging produces relatively accurate predictions.^18^ RSF requires minimal data assumptions and automatically accounts for non-linear effects, complex interactions, and high correlations among predictors.^18^ For example, the right side of Figure S1 depicts an interaction among TMS and age because the split of age depends on the split of TMS. The left side of Figure S1 depicts a non-linear effect of TMS because the second TMS split is conditional on the first TMS split. TMS also has a main effect because the root node split (Node 1) is not conditional on any other variable (including TMS itself).

Prediction error for a RSF is measured by 1 − *C*, where *C* is Harrell’s concordance index,^19^ widely used in survival analysis. *C* indexes the ability to rank the observed time to diagnosis based on the predicted risk of diagnosis, accounting for censoring. Consider pairs of people with observed time to diagnosis or censoring, and their associated estimated probability of diagnosis based on RSF (or the Cox model). A pair is concordant if the person with the shorter observed time has a higher predicted probability of diagnosis than the person with the longer observed time (some pairs, such as two people with censoring, cannot be compared and they are omitted from the analysis). *C* is the proportion of concordant pairs for a particular time period, with *C* = 1 denoting perfect concordance (and *C* = 0 indicating worst possible agreement). It follows that prediction with no error is indicated by 1 − *C* = 0.

After averaging over the trees, two methods were considered for evaluating the merit (strength) of a predictor: variable importance and minimal depth. Variable importance compares the prediction error of normally grown trees (Figure S1) to that of trees in which the daughter nodes are randomly assigned. For example, in Figure S1 after a predictor and cut-value are determined for a node, the cut rules (e.g., TMS ≤ 11, TMS > 11) are randomly assigned to the left and right. If a predictor has little merit in predicting survival, the random assignment will have little effect relative to the normal assignment. The discrepancy between the prediction error (= 1 − *C*) of the normal and random-node trees will be small, and so will the variable importance index. On the other hand, when the predictor has much merit, the discrepancy will be large, and so will the variable importance. Thus, higher values of variable importance indicate greater merit. ^20^

Minimal depth indexes how close to the root node a predictor tends to be among the trees.^21^ The predictor used to split the root node (Node 1) is the most important in prediction for a tree, and merit decreases for variables as they appear in deeper nodes. For example, in Figure S1, TMS has greater merit than age because the first appearance of the former is at a lower depth than the latter. Minimal depth indexes the first-appearance depth of a variable across all trees. It follows that smaller values of minimal depth indicate greater merit.

Interaction analysis was carried out by randomly assigning left and right split-value rules for two variables. For example, in Figure S1 after determining that TMS and age will be the predictors on the right side of the tree, with split-values 11 and 51, respectively, the rules are randomly assigned for both variables (this is in contrast to variable importance for which random assignment is for only one variable). The prediction error under the normal assignment (Figure S1) is compared to that under random assignment and averaged over all trees. To the extent an interaction is important, there will be a discrepancy between the prediction error under the two scenarios, and the size of the interaction importance will be large in absolute value (interaction importance can be a positive or negative number). Conversely, if the interaction is negligible, the interaction importance tends to be close to zero. Table S1 shows the partial results of the interaction analysis. As discussed in the text, the interaction importance for CAG × age is the largest, indicating this is the most important interaction to consider.

The log cumulative hazard was chosen as the outcome for the Figure 2 graphs (see main text). The reason for the log transformation is that in the Cox model, the log hazard has a linear relationship with the predictors (see Equation 1 of the main text).

All analysis was carried out using the R program for statistical computing.^22^ The randomForestSRC package was used for the RSF analysis,^23^ and the pec package was used for the cross-validation.^24^

**Table S1.** Interaction importance for the random survival forest interaction analysis based on the 8-predictor set.

| **Interaction** | **Effect** |
| --- | --- |
| CAG × Age | 0.0151 |
| CAG × TMS | 0.0120 |
| CAG × DCL | 0.0105 |
| TMS × DCL | 0.0065 |
| SDMT × DCL | 0.0045 |
| CAG × Inter | 0.0042 |

The effect size is computed from the joint randomization of a pair of variables in the random survival forest analysis. Larger values indicate greater predictive strength of the interaction. Six largest values are shown. CAG, cytosine-adenine-guanine expansion; Age, age at study entry; TMS, total motor score; DCL, diagnostic confidence level; SDMT, Symbol Digit Modalities Test; Inter, Stroop interference test.

**Variability of the Total Motor Score in PREDICT-HD**

**Baseline variability:** As mentioned in the text, the UHDRS total motor score (TMS) had substantial variability, and there was extensive overlap among the TMS distributions conditioning on the levels of the diagnostic confidence level (DCL). Figure S2 shows boxplots and observed values of TMS by DCL level at baseline in PREDICT-HD. The TMS scores were jittered horizontally (small random error added to DCL) in order to minimize overlapping cases. No participant had DCL = 4 at baseline, which explains the absence of this stratum in the figure.

**All time points:** To gain additional insight into TMS variability and its relation to DCL, the boxplots and individual values were constructed for all time points in PREDICT-HD shown in Figure S3. Most participants had longitudinal data and contributed to more than one value in the figure.

**Figure S2**. Boxplots and baseline values of total motor score (circles) as a function of diagnostic confidence level.


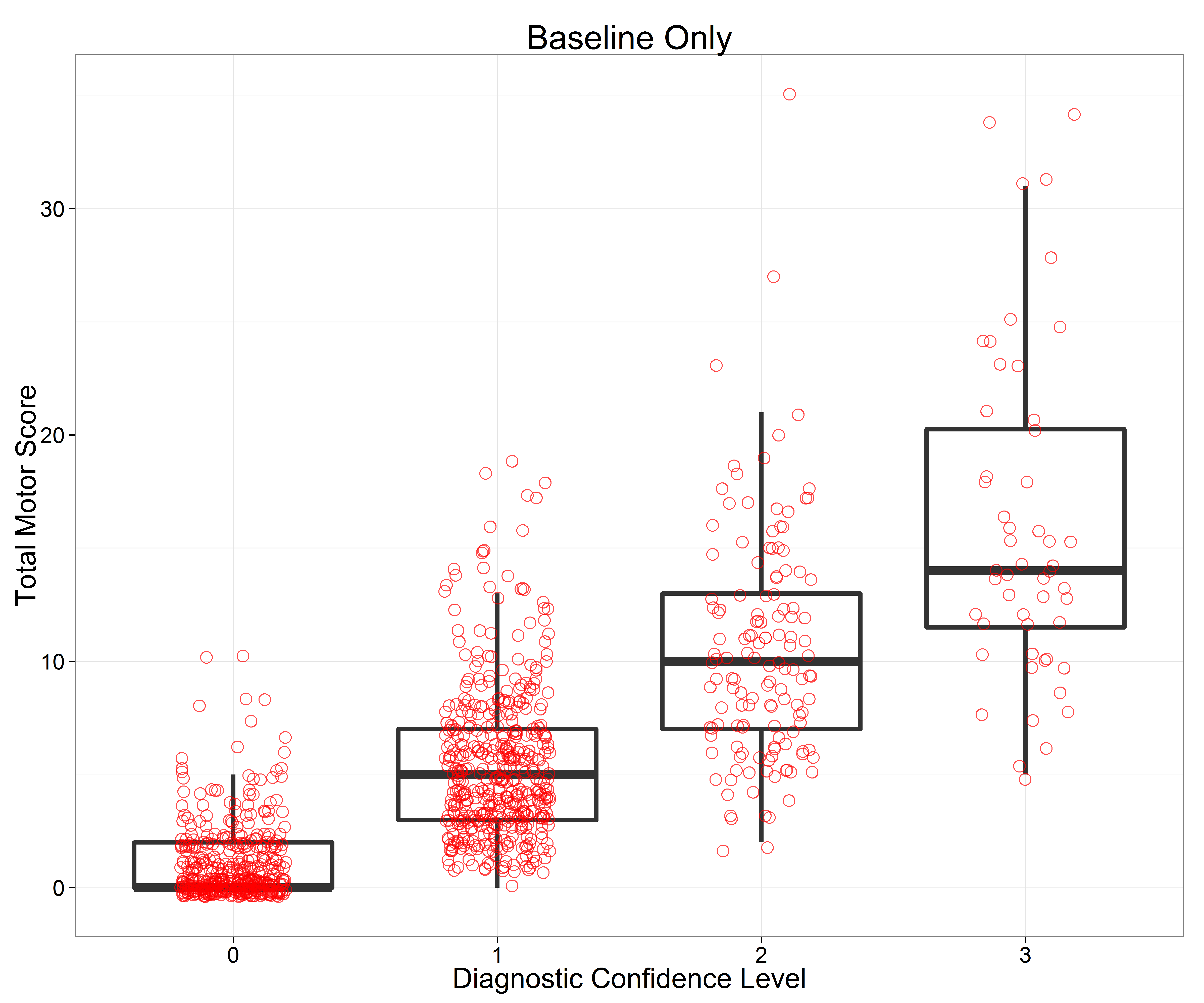


**Figure S3**. Boxplots and values of total motor score (circles) as a function of diagnostic confidence level: all times.


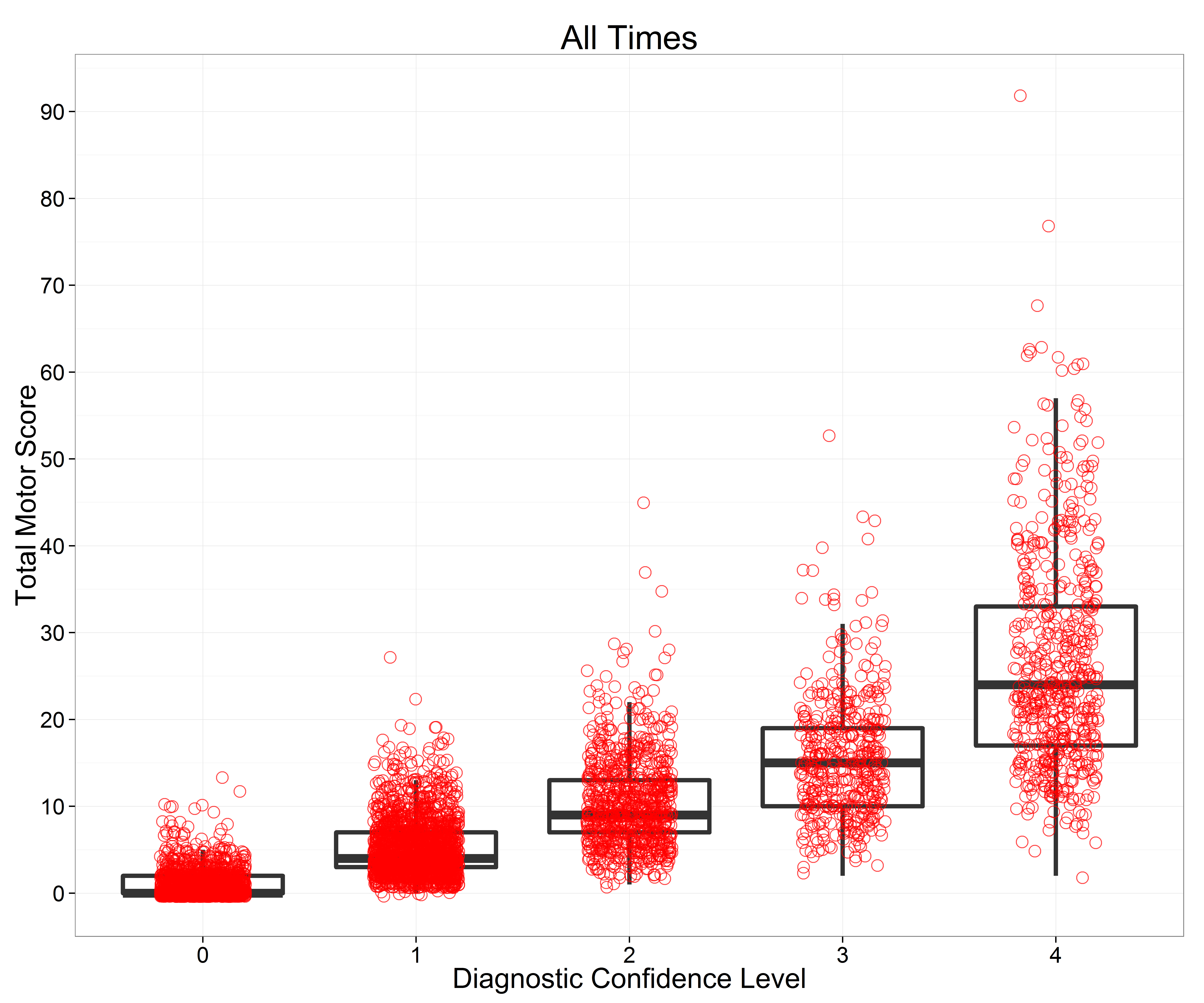


**Supplemental material references**

1. Huntington Study Group. Unified Huntington's Disease Rating Scale: reliability and consistency. Mov Disord 1996;11:136-142.

2. Rowe KC, Paulsen JS, Langbehn DR, et al. Self-paced timing detects and tracks change in prodromal Huntington disease. Neuropsychology 2010;24:435-442.

3. Wechsler D. Manual for the Wechsler Adult Intelligence Scale - Revised, New York, NY, Psychological Corporation, 1981.

4. Lezak MD, Howieson D, Loring D. Neuropsychological assessment, 4th ed, New York, Oxford University Press, 2004, pp 1016.

5. Smith A. Symbol Digit Modalities Test (SDMT) Manual (Revised), Los Angeles, CA, Western Psychological Services, 1982.

6. Stroop JR. Studies of interference in serial verbal reactions. J Exp Psychol 1935;18:643-662.

7. Golden C. Stroop Color and Word Test: Cat. No. 30150M; a Manual for Clinical and Experimental Uses, Chicago, IL, Stoelting, 1978.

8. Doty RL, Shaman P, Kimmelman CP, Dann MS. University of Pennsylvania Smell Identification Test: a rapid quantitative olfactory function test for the clinic. Laryngoscope 1984;94:176-178.

9. O'Rourke JJ, Beglinger LJ, Smith MM, et al. The Trail Making Test in prodromal Huntington disease: contributions of disease progression to test performance. J Clin Exp Neuropsychol 2011;33:567-579.

10. Reitan R. Validity of the trail making test as an indicator of organic brain damage. Percept Mot Skills 1958;8:271-276.

11. Johnson SA, Stout JC, Solomon AC, et al. Beyond disgust: impaired recognition of negative emotions prior to diagnosis in Huntington's disease. Brain 2007;130:1732-1744.

12. Ekman P, Friesen WV. Measuring Facial Movement. Environmental Psychology and Nonverbal Behavior 1976;1:56-75.

13. Aylward EH, Nopoulos PC, Ross CA, et al. Longitudinal change in regional brain volumes in prodromal Huntington disease. J Neurol Neurosurg Psychiatry 2011;82:405-410.

14. Derogatis L. Administration, scoring & procedures manual-I for the revised version and other instruments of the psychopathology rating scale series, Baltimore, MD, John Hopkins University, 1977.

15. Grace J, Malloy P. Frontal systems behavior scale: professional manual, Lutz, FL, Psychological Assessment Resources, Incorporated, 2000, pp 109.

16. Beck A, Steer R, Brown G. Beck Depression Inventory-II (BDI-II): Manual for Beck Depression Inventory-II, Pearson, 1993.

17. Breiman L. Random forests. Machine Learning 2001;45:5-32.

18. Chen X, Ishwaran H. Random forests for genomic data analysis. Genomics 2012;99:323-329.

19. Harrell FEJ, Califf RM, Pryor DB, Lee KL, Rosati RA. Evaluating the yield of medical tests. JAMA 1982;247:2543-2546.

20. Ishwaran H, Kogalur U, Blackstone E, Lauer M. Random survival forests. Ann Appl Stat 2008;2:841-860.

21. Ishwaran H, Kogalur UB, Gorodeski EZ, Minn AJ, Lauer MS. High-Dimensional Variable Selection for Survival Data. J Am Stat Assoc 2010;105:205-217.

22. R Development Core Team. R: A language and environment for statistical computing. R Foundation for Statistical Computing. Vienna, Australia: R Foundation for Statistical Computing; 2014.

23. Ishwaran H. Variable importance in binary regression trees and forests. Electron J Stat 2007;1:519-537.

24. Mogensen UB, Ishwaran H, Gerds TA. Evaluating Random Forests for Survival Analysis Using Prediction Error Curves. J Stat Softw 2012;50:1-23.
